# Multidisciplinary management of thyroid eye disease (TED) in patients with latent tuberculosis or chronic hepatitis B

**DOI:** 10.1186/s13044-025-00283-2

**Published:** 2026-01-29

**Authors:** John S. Vekinis, Jonathan Lee, Vickie Lee, Ahmad Aziz, Rajni Jain, Claire Feeney, Vassiliki Bravis

**Affiliations:** 1 Western Eye Hospital, Imperial College Healthcare NHS Trust, London, UK; 2 Department of Surgery & Cancer, General Surgery, Faculty of Medicine, Imperial College London, London, UK; 3 Department of Metabolic Medicine, St Mary’s Hospital, Imperial College Healthcare NHS Foundation Trust, London, UK; 4 Department of Metabolism, Faculty of Medicine, Digestion and Reproduction, Imperial College London, London, UK

**Keywords:** TED, Immunosuppression, IVMP, Tuberculosis, MDT, Hepatitis, GO-QOL

## Abstract

**Background:**

Intravenous methylprednisolone (IVMP) is a first-line treatment for active moderate-severe or sight-threatening thyroid eye disease (TED), but the presence of systemic infections such as latent tuberculosis (TB) or chronic hepatitis B virus (HBV) act as relative contraindications due to the risk of reactivation. There is limited data to guide clinicians in this high-risk group.

**Methods:**

A retrospective case series was conducted involving five TED patients with relative contraindications to immunosuppression. Each patient included had a EUGOGO severity of moderate-to-severe. Multidisciplinary team (MDT) input guided treatment planning. Patients either received IVMP with or without mycophenolate mofetil (MMF) alongside management of their systemic infection or were observed. TED outcomes, infection status, and adverse events were monitored.

**Results:**

Three patients had latent TB and received isoniazid ± rifampicin prophylaxis with two having conservative TED management, due to high-risk features of their TB, and one receiving IVMP with MMF. Two patients had chronic HBV and underwent IVMP therapy with tenofovir or entecavir. No systemic infection progression or adverse events were observed during follow-up. All treated patients showed improvement in Clinical Activity Score (CAS) and improved or stable Graves’ Ophthalmopathy Quality of Life (GO-QOL) scores.

**Conclusions:**

Immunosuppression can be safely initiated in TED patients with systemic infections using a multidisciplinary approach. Early screening, judicious patient selection, coordinated care, and prophylactic treatment are key to balancing efficacy with risk mitigation.

## Background

Thyroid eye disease (TED) is a disfiguring and potentially sight-threatening autoimmune condition most associated with Graves’ hyperthyroidism. It is characterised by inflammation and expansion of the orbital tissues, resulting in proptosis, diplopia, and, in severe cases, optic neuropathy. Current European Group on Graves’ Orbitopathy (EUGOGO) guidelines recommend intravenous methylprednisolone (IVMP) with or without mycophenolate mofetil (MMF) as first-line treatment for moderate-to-severe TED and IVMP without MMF for sight-threatening TED [1]. However, EUGOGO guidelines do not comment on the appropriateness of administering IVMP in patients with latent tuberculosis (TB) infection, and only recent viral hepatitis B (HBV) infection is listed as an absolute contraindication with no recommendation for patients with chronic infection. Both latent TB and chronic hepatitis have well established risks of reactivation secondary to IVMP and being aware of the TB and hepatitis status of patients prior to IVMP can avoid adverse outcomes [2, 3].

With uncertainty over the importance of latent infections, treatment can be delayed or withheld due to concerns regarding infectious complications. However, in sight-threatening TED, this approach risks progression of TED and potential irreversible visual loss. Reports of TED management in this group are limited, and most clinical trials of novel biological treatments exclude patients with chronic infections. Though evidence from other specialties suggests that with appropriate screening, antiviral or anti-tuberculous prophylaxis, and multidisciplinary oversight, immunosuppressive treatment can be administered safely [4, 5].

This study presents a retrospective case series of five patients with moderate-to-severe TED and concurrent latent TB or chronic HBV infection. All were managed through a multidisciplinary team (MDT) including endocrinology, ophthalmology, infectious diseases, and hepatology. We aimed to describe the outcomes of MDT led treatment of moderate-to-severe TED in this cohort. This included patients receiving IVMP based immunosuppression, those in whom immunosuppression was considered but deferred due to infectious risk and those who did not have IVMP considered due to inactive disease. These findings may help inform practical treatment pathways for clinicians managing TED in patients with co-existing infectious risk factors.

## Methods

Data on all patients with moderate-to-severe active or inactive TED and systemic infection (latent TB or HBV) undergoing treatment at the Imperial Healthcare TED MDT clinic was collected over a five-year period between 2020 and 2025. All patients had endocrine, ophthalmology and infectious disease consultant review. Inclusion criteria included moderate-to-severe active or inactive TED along with latent TB or chronic HBV. Moderate-to-severe TED was defined as patients without sight-threatening TED whose eye disease has sufficient impact on daily life to justify the risks of immunosuppression (if active) or surgical intervention (if inactive), and active TED was defined as a clinical activity score (CAS) ≥ 3 as per EUGOGO guidelines [1]. Both patients who received systemic immunosuppression and did not were included, as the purpose of the study was to document MDT assessment, treatment decision-making, and clinical outcomes in this high-risk population. Exclusion criteria included lack of follow-up data defined as less than 6 months in duration.

Data collected from electronic health records included demographics, thyroid and infection status, thyroid stimulating hormone receptor antibody (TSHR-Ab) titre at diagnosis, treatment outcomes, CAS [6], Gorman [7] and Graves’ ophthalmopathy quality of life (GOQOL) scores [8]. Outcomes from full ophthalmic assessment was also collected to assist with classification of TED severity, this included visual acuity, intraocular pressure, anterior and posterior segment examination, orthoptic assessment, amount of lid retraction and degree of exophthalmos [1]. GO-QOL was evaluated in both the visual and appearance subdomains at baseline and follow-up.

All patients underwent screening for latent TB and HBV as per institutional protocols prior to initiating immunosuppression with TB Elispot or QuantiFERON Gold, and HBV core total and surface antibody testing respectively. Infection prophylaxis (isoniazid with or without rifampicin, tenofovir or entecavir) was started in collaboration with infectious disease or hepatology teams and was commenced 0–2 months prior to any immunosuppression if immunosuppression was indicated. The decision to commence TED treatment was based on assessment by the MDT and decisions were made on a case-by-case basis. If immunosuppression was commenced, it was with the EUGOGO first line treatment of IVMP (500 mg weekly for six weeks, then 250 mg weekly for six weeks) with or without MMF (up to 1 g twice daily for six months). All patients were monitored for infection reactivation, liver function, and adverse events.

This retrospective audit was registered and approved by Imperial College Healthcare National Health Service (NHS) Trust, London. Data were anonymised and confidentiality was maintained throughout. This audit adhered to the tenets of the Declaration of Helsinki.

## Results

Five patients were included, three with latent TB and two with chronic HBV infection. Two of the five patients were hyperthyroid at the time of TED treatment decision, and these patients were treated with carbimazole titration to achieve a euthyroid state. The remaining three patients were euthyroid at TED treatment decision. All patients had an established diagnosis of Grave’s disease. Table 1 summarises the patient characteristics and management plans.

**Table 1:** Characteristics and management of TED patients with chronic infections acting as relative contraindications to immunosuppression

Patient Number	Age at treatment decision	Gender	TSHR-Ab titre at diagnosis (IU/L)	Infection	Thyroid status at treatment decision	Follow up period (days)	Time from TED diagnosis to treatment decision (months)	TED management	Infection management
TB1	39	F	1.8	Latent TB	Euthyroid	161	23	IVMP and MMF	Isoniazid for six months
TB2	77	M	6.4	Latent TB	Euthyroid	218	14	None	Isoniazid and rifampicin for six months
TB3	56	F	3.6	Latent TB	Hyperthyroid	252	58	None	Isoniazid and rifampicin for six months
HB1	59	M	10	Chronic HPB	Euthyroid	219	0	IVMP and MMF	Tenofovir prophylaxis
HB2	52	F	10.3	Chronic HPB	Hyperthyroid	255	1	IVMP	Entecavir prophylaxis

TB: Tuberculosis. HB/HPB: hepatitis B. TSHR-Ab: thyroid stimulating hormone receptor Antibody. TED: thyroid eye Disease. IVMP: intravenous Methylprednisolone. MMF: mycophenolate mofetil

**Table 2:** Clinical scoring results before and after TED management decisions in patients with chronic infections acting as relative contraindications to immunosuppression

Patient Number	CAS		Gorman Score		GOQoL Visual Score		GOQoL Appearance Score	
	Pre	Post	Pre	Post	Pre	Post	Pre	Post
TB1	3	2	1	3		7.14		18.75
TB2	0	1	3	0	100	100	100	100
TB3	3	2	1	0	100	100	31.25	37.5
HB1	3	0	0	0	100	100	100	100
HB2	6	4	2	2	0	58.33	0	0

TB: Tuberculosis. HB: hepatitis B. CAS: clinical activity Score. GOQOL VF/App, graves’ ophthalmopathy quality of life visual Function/Appearance

TED clinical activity, subjective diplopia and quality-of-life before and following TED management decisions are summarised in Table 2. CAS improved in all patients receiving immunosuppression, with TB1 improving from three to two, HB1 from three to zero, and HB2 from six to four. Gorman diplopia scores remained stable or improved in all cases, apart from TB1, who increased from one pre-treatment to three post-treatment. GOQoL visual and appearance scores was stable or improved in four out of five patients with one patient unfortunately not having pre-treatment GOQoL scores. HB2, who had the highest baseline CAS, showed partial visual improvement but a persistently low GOQoL appearance score. Two patients with TB did not receive immunosuppression (TB2 and TB3) due to a chronic cough and a new diagnosis of TB during pre-IVMP assessment, both remained clinically stable and had reduced Gorman scores following observation. All patients had stable liver functions, and no progression of systemic infection or adverse events were observed during follow-up.

## Discussion

This study describes the outcomes of a cohort of patients with active moderate-severe TED with relative contraindications to immunosuppression due to systemic chronic infection, and highlights the central role of the multidisciplinary team in collaborative decision making on risk stratification.

None of the patients in our cohort experienced reactivation of their infection following immunosuppressive therapy. Two patients with chronic HBV received IVMP after commencing antiviral prophylaxis and remained biochemically and clinically stable throughout follow-up. Similarly, one patient with latent TB was treated with IVMP and MMF following six months of isoniazid therapy. Notably, British and American TB guidelines do not advocate for prophylactic TB treatment in all cases of latent TB undergoing IVMP despite corticosteroids increasing the risk of TB reactivation 2.8- to 7.7-fold [3–5, 9]. Other authors have suggested it is reasonable to offer prophylactic TB treatment in high-dose corticosteroid therapy in a similar fashion to the well-established guidance to commence TB prophylaxis when starting anti-TNF-alpha treatment [10, 11]. Our cohort followed similar principles to TB prophylaxis in patients commencing anti-TNF-alpha treatment, all TB-positive patients receiving prophylaxis for 3–6 months under infectious disease guidance and none developed systemic symptoms, radiographic changes, or liver enzyme derangement. Our findings support the use of prophylactic TB treatment in the case of latent TB to safely reduce the risk of reactivation [12]. Notably, two TB patients not treated with immunosuppression remained stable and showed improved TED-related scores despite treatment with observation alone. This suggests that a conservative approach remains valid for selected cases without progressive disease.

HBV reactivation is another recognised risk of immunosuppressive therapy. It can occur in inactive carriers and resolved infections, with reactivation leading to sequalae ranging from asymptomatic viral replication, hepatitis flares, or fulminant liver failure [2]. Corticosteroids are a well-established cause of HBV reactivation as they both immunosuppress and increase the expression of HBV through a glucocorticoid-responsive element [13]. In TED, IVMP regimens typically span 12 weeks and involve cumulative steroid doses exceeding 4 g, placing them in the high-risk category for HBV reactivation and warranting prophylactic treatment [14]. Both HBV patients in our study received guideline-based antiviral prophylaxis, one with tenofovir and one with entecavir, and remained stable. This reflects current recommendations though there are no significant case series in the literature describing the management of active TED in patients with chronic HBV [15]. Our study adds further support to the safe use of IVMP in chronic HBV when prophylactic antiviral therapy is employed.

Study limitations include the small sample size and as a retrospective analysis, our findings may be subject to selection and documentation bias. Some relevant patient characteristics were not recorded in patient notes such as ductions quantification and measurement of eyelid apertures, this data could be collected in any future prospective study. As this is a case series, we unable to perform statistical analysis of the observed outcomes. Nonetheless, our results represent one of the most detailed TED-focused reports in this subgroup and provide valuable clinical data in a previously underrepresented population.

## Data Availability

The datasets generated and/or analysed during the current study are not publicly available due being healthcare data requiring approval to access from Imperial College Healthcare National Health Service (NHS) Trust. The data may be available from the corresponding author on reasonable request.
